# Behavioral Responses of the Common Bed Bug, *Cimex lectularius*, to Insecticide Dusts

**DOI:** 10.3390/insects8030083

**Published:** 2017-08-08

**Authors:** John L. Agnew, Alvaro Romero

**Affiliations:** Department of Entomology, Plant Pathology and Weed Science, New Mexico State University, Las Cruces, NM 88003, USA; agnewj@nmsu.edu

**Keywords:** *Cimex lectularius*, insecticide dust, diatomaceous earth, behavioral responses, avoidance, video recording

## Abstract

Bed bugs have reemerged recently as a serious and growing problem not only in North America but in many parts of the world. These insects have become the most challenging pest to control in urban environments. Residual insecticides are the most common methods used for bed bug control; however, insecticide resistance limits the efficacy of treatments. Desiccant dusts have emerged as a good option to provide a better residual effect for bed bug control. Several studies have focused on determining the efficacy of dust-based insecticides against bed bugs. However, behavioral responses of bed bugs to insecticide dusts could influence their efficacy. The behavioral responses of bed bugs to six insecticide dusts commonly used in the United States were evaluated with an advanced video tracking technique (Ethovision). Bed bugs took longer to make first contact with areas treated with the diatomaceous earth (DE)-based products MotherEarth D and Alpine than pyrethroid, pyrethrins or silica gel based products, DeltaDust, Tempo 1% Dust and CimeXa, respectively. Lower visitation rates of bed bugs were recorded for areas treated with MotherEarth D, Alpine and CimeXa than that of DeltaDust, Tempo 1% Dust, and Tri-Die Silica + Pyrethrum Dust. Bed bugs spent less time in areas treated with Tri-Die Dust, CimeXa, Alpine, and MotherEarth D than DeltaDust and Tempo 1% Dust, and they exhibited a reduction in locomotor parameters when crawling on areas treated with CimeXa and Alpine. The implications of these responses to bed bug control are discussed.

## 1. Introduction

Bed bugs have emerged as a serious pest of worldwide concern [1,2]. Bed bug infestations pose significant social, economic, and public health burdens [3,4,5]. These insects are difficult to control due to their nocturnal habits and cryptic behavior, a limited number of available active ingredients for control, and the low efficacy of residual insecticides [6,7]. Although integrated pest management (IPM) approaches that incorporate chemical and nonchemical methods have been proposed as an effective bed bug management [8,9,10], insecticide treatments continue to be the most common tool for bed bug control among pest management professionals [1,11]. Insecticide sprays are often applied extensively to bed bug aggregations or potential hiding places [12]; however, efficacy of insecticides is hampered by the development of insecticide resistance among bed bug populations [13,14,15,16]. Desiccant dusts have a long history of being used for pest management [17]. Silicon dioxide-based desiccants (e.g., diatomaceous earth [DE] and silica gel) are popular dusts because of their low mammalian toxicity and long residual insecticidal effects on crawling insects [17]. Diatomaceous earth and silica gel insecticide dusts work primarily by damaging the waxy cuticle of the insect through sorption or abrasion that can result in desiccation and death [18,19]. Insecticide dusts are typically applied to areas such as behind outlets and switch plates, beneath baseboards and carpet edges, or along inner frameworks of couches and box springs [12]. Insecticide dusts have also been used as perimeter treatments around beds and other furniture, along baseboards, and in voids [20]. They are considered a low-cost, long-term residual treatment option for low-income, multiunit housing buildings [20,21].

Adult and nymph bed bugs feed on the blood of humans and other vertebrates for brief time periods. When not feeding, bed bugs spend their time in concealed aggregation areas usually in close proximity to hosts [6]. Bed bugs are usually found assembled along mattress and box spring seams, as well as inside cracks, crevices, edges of furniture, baseboards, electric outlets, and other similar locations [22]. Because bed bugs do not reside on living hosts, they repeatedly need to leave their harborages to seek out their host for a blood meal [23]. It is during this time that, while searching for a blood meal or returning to their harborages, insects might come in contact with insecticide residues.

Insecticides can influence insect behavior by disrupting the normal function of its sensory or central nervous system [24]. For example, bed bugs avoid resting in areas with pyrethroid residues, which could reduce their exposure to insecticides [25]. Insecticides also affect several aspects of bed bug biology, including locomotion, feeding, mating, oviposition, fecundity, and development [25,26,27,28]. These sublethal effects could have detrimental consequences on bed bug populations over time. Studying behavioral responses of bed bugs to insecticides will ultimately provide a better understanding of the overall impact of insecticide treatment in bed bug management programs [25]. Although insecticide dusts have been perceived as a good option for bed bug control [29,30,31], data from previous studies on other insects indicate that some dusts might have repellent effects [32,33,34]. These effects could decrease the efficacy of insecticide dusts against bed bugs. In this study, we aim to quantify the behavioral responses of bed bugs to six common insecticide dust products with a modern video tracking technique. We discuss the impact of avoidance responses on bed bug control practices.

## 2. Materials and Methods

### 2.1. Insects

Adult bed bugs were obtained from a colony maintained at 25 °C, 50 ± 5% relative humidity, and a photoperiod of 12 h:12 h (light:dark). This colony was originally established from bed bugs collected in 2008 from an apartment in Jersey City, NJ, USA. This population was determined to be resistant to deltamethrin, following a method proposed by Romero et al. [13] (discriminating doses of 0.13 mg/cm^2^ technical grade deltamethrin; 0% mortality in 20 third to fifth instar nymphs). In the laboratory, the insects were fed defibrinated rabbit blood (Quad Five, Ryegate, MT, USA), heated to 37 °C with a circulating water bath, through a Parafilm-membrane feeder [35]. Evaluations began 8 to 10 days after adult emergence; the insects had been fed as adults three days before the initiation of the experiment.

### 2.2. Insecticide Dusts

The trade names, active ingredients, label rates of application, and manufacturer information for six dust insecticides, representing different classes of insecticides, are as follows, pyrethroid: DeltaDust (0.05%, deltamethrin, Bayer Environmental Science, RTP, NC, USA), Tempo 1% Dust (1% cyfluthrin, Bayer Environmental Science, RTP, NC, USA); pyrethrins and silica gel: Tri-Die Silica + Pyrethrum Dust (1% pyrethrins, 10% piperonyl butoxide, and 40% amorphous silica, BASF Corporation, St. Louis, MO, USA); silica gel: CimeXa (92.1% amorphous silica gel, Rockwell Labs Ltd., North Kansas City, MO, USA); neonicotinoid and inorganic: Alpine (0.25% dinotefuran, 95% DE, BASF Corporation, St. Louis, MO, USA); and inorganic: MotherEarth D (100% DE, BASF Corporation, St. Louis, MO, USA).

### 2.3. Arenas

**Figure 1:**
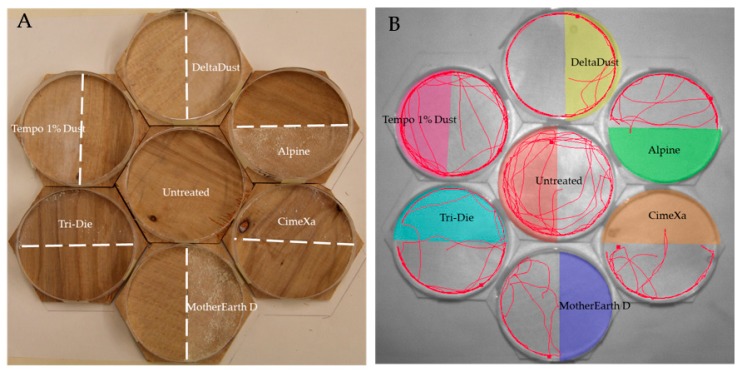
View of experimental arenas: (**A**) Plywood hexagons included halves treated with insecticide dusts. An untreated hexagon was always included in each replicate to act as control; (**B**) The activity of individual insects in each hexagon was tracked with EthoVision^®^ XT software to generate behavioral parameters (tracks of individual bed bugs during 5-min recordings).

Behavioral responses were tested in seven hexagon arenas (six treated with dust and one control) placed in a honeycomb pattern. This pattern was selected to accommodate for the limited viewing field of the camera used (Figure 1). The hexagons (length of sides = 5.4 cm, total area = 62.2 cm^2^) were made of plywood and were never reused. Placed on top of each hexagon was a ring made from a petri dish plate (diameter = 9 cm) to restrain the bugs in the arena. The internal ring walls were coated with fluoropolymer resin to prevent insects from climbing and escaping. Each arena was then divided in half, with one half being the treated zone and one being the untreated zone. The control arena was also divided in half, in which one half was randomly selected to gather activity variables so that comparisons with the other six treated arenas could be made. To determine the orientation of the split, a cardinal point system was used for north, east, south and west, and positions were randomized in each replicate. The amount of dust required to treat each half of the hexagons was calculated based on the label rate. The mean weights (± SE) of the products applied were 0.447 ± 0.007, 0.704 ± 0.008, 0.534 ± 0.007, 0.526 ± 0.005, 1.026 ± 0.009, and 1.566 ± 0.009 mg/cm^2^ for DeltaDust, Tempo 1% Dust, Tri-Die Dust, CimeXa, Alpine, and MotherEarth D, respectively.

Dust was distributed evenly on the plywood surfaces with a painting brush. A piece of plexiglass, fitted to the petri dish arena, was placed along the midline during application to prevent contamination of the untreated half. The hexagons were shifted in a counterclockwise manner between each replicate, with the center hexagon moving to the lower right space, the bottom hexagon moving into the center space, and the remaining hexagons moving respectively counterclockwise. Individual bed bugs were acclimated to the environment by restricting them in a piece of plastic tubing which was placed in the untreated area for 5 min. Insects were released by lifting up the tubing in each arena. Approximate simultaneous release was achieved by pinning fishing line to each of the plastic tubes and pulling them all up at the same time. In total, 20 replicates (10 males and 10 females) were used along with a common control group for each. Bioassays were conducted under ambient temperature (25 ± 2 °C) and relative humidity (40 ± 10%) during the first three hours into the scotophase, a time in which bed bugs display enhanced locomotor activity [36].

### 2.4. Tracking Activity

A near-infrared (NIR) camera (series acA1300-60 gm NIR camera, Basler^®^ ace; Exton, PA, USA), outfitted with a lens (C-mount 4–8 mm varifocal megapixel CCTV lens, model# H2Z0414C-MP, Computar^®^; Torrance, CA, USA) and (infrared) IR filter (Infrared 850 light filter, Heliopan^®^, North White Plains, NY, USA) was used to record bed bug activity in the arenas under dark conditions. The camera was positioned approximately 58 cm directly above the center arena. Light for the recordings was provided by two IR illuminators (AT-8SB 850 mm, 130°, AXTON^®^, North Salt Lake, UT, USA). EthoVision XT version 11.5 software (Noldus Information Technology Inc. Leesburg, VA, USA, [37]) was used to capture video images and to track the bed bugs during 5-min bioassays. EthoVision XT virtually facilitates the division of each arena into two equal zones known as “treated” and “untreated” (Figure 1B). Multiple variables were calculated from dust-treated arenas: elapsed time until first visit to the treated zone, number of visits to the treated zone, percent of time spent in the treated zone, and distance traveled and velocity in treated zone. The same variables were calculated from the activity of bed bugs recorded in control arenas.

### 2.5. Forced Exposure Assays

Groups of 10 bed bugs (three replicates) were forcibly exposed to the dusts for, approximately, the average time the insects spent crawling on each dust, calculated from the 5-min period bioassays described in the behavioral study: 15 s, 1 min 9 s, 1 min 14 s, 1 min 31 s, 2 min 8 s, 2 min 12 s, for DE, Alpine, CimeXa, Tri-Die Dust, Tempo, DeltaDust, respectively. Bed bugs were confined to treated halves of hexagons with the same dimensions used in the behavioral assays. Confinement to treated areas was achieved by fitting a piece of plexiglass into the arena along the center line. Bed bugs were removed from treated areas after each exposure time and placed in clean areas to record mortality at Day 4.

### 2.6. Data Analysis

Elapsed time until the first visit to the treated areas and the percent of time spent in the treated areas were analyzed with the nonparametric Mann–Whitney test using Minitab [38]. The number of visits to the treated zones, the distance, and the velocity were analyzed using one-way analysis of variance (ANOVA). The many-to-one comparison was done with Dunnett’s test for mean separation [38].

## 3. Results

**Figure 2:**
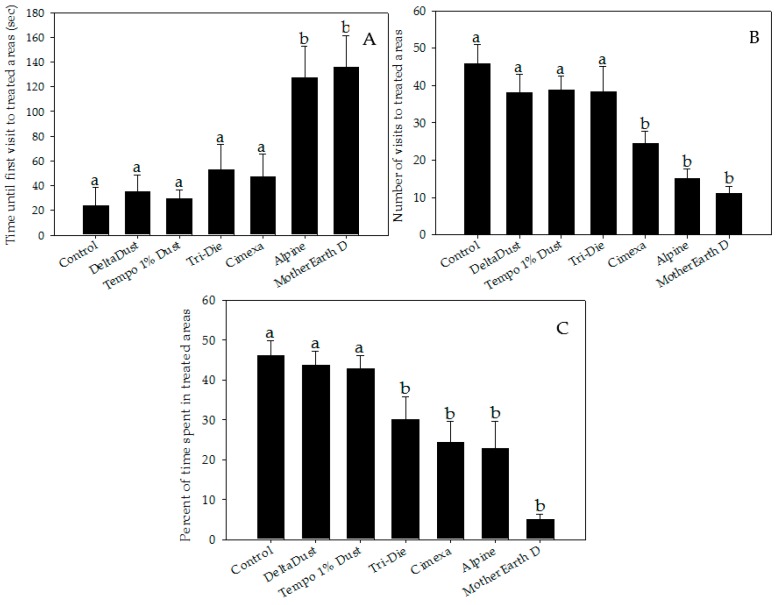
Behavior parameter measurements of bed bugs interacting with areas treated with insecticide dusts during 5-min recordings: (**A**) elapsed time until first visit; (**B**) number of visits; and (**C**) percent of time spent. Data are presented as means ± SE. Bars that have the same letter as the control indicate that there is no significant statistical difference between the given dust treatment and control arena (One-way ANOVA, Dunnett’s test, *p* > 0.05; Mann–Whitney W test, *p* > 0.05).

Bed bugs significantly avoided areas treated with CimeXa, Alpine, Tri-Die Dust, and MotherEarth D (Figure 1 and Figure 2, and videos in supplementary file). When compared with that of control arenas, the elapsed time until the bed bug’s first visit to the treated area was significantly longer when the area was treated with Alpine (mean times = 127.6 ± 24.9 s vs. 23.7 ± 14.9 s, respectively; *W* = 301.0, *p* < 0.05) or MotherEarth D (mean times = 136.1 ± 25.3 s vs. 23.7 ± 14.9 s, respectively; *W* = 279, *p* < 0.05) (Figure 2A).

**Figure 3:**
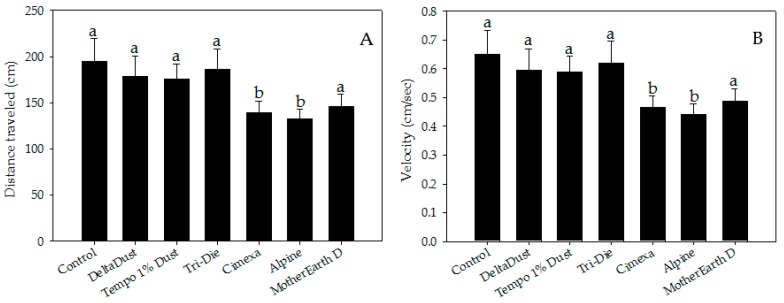
Locomotor parameter measurements of bed bugs in areas treated with insecticide dusts: (**A**) distance traveled and (**B**) velocity. Data are presented as means ± SE. Bars that have the same letter as the control indicate that there is no significant statistical difference between the given dust treatment and control arena (One-way ANOVA, Dunnett’s test, *p* > 0.05).

Insecticide dust avoidance was also reflected by lower visitation rates of bed bugs to treated areas (Figure 2B). Although approximately half of bed bugs visited control halves (mean = 45.8 ± 5.1%), a significantly lower proportion of bed bugs visited areas treated with CimeXa (24.5 ± 3.1%, *T* = −3.451, df = 6, *p* < 0.05), Alpine (15.0 ± 2.6%, *T* = −4.994, df = 6, *p* < 0.05), and MotherEarth D (11.0 ± 1.9%, *T* = −5.644, df = 6, *p* < 0.05) (Figure 2B). Pair-wise analysis showed that bed bugs spent significantly less time in areas treated with the following dust insecticides than in control halves: CimeXa (24.4 ± 5.1% vs. 46.0 ± 3.8%; *W* = 525.5, *p* < 0.05), Alpine (22.9 ± 6.8% vs. 46.0 ± 3.8%; *W* = 506.0, *p* < 0.05), Tri-Die Dust (30.1 ± 5.7% vs. 46.0 ± 3.8%; *W* = 510.5, *p* < 0.05), and MotherEarth D (5.0 ± 1.3% vs. 46.0 ± 3.8%; *W* = 587.0, *p* < 0.05) (Figure 2C). The distance traveled by bed bugs in treated areas was also affected by the type of insecticide dust used (Figure 3A). Bed bugs in areas treated with CimeXa (mean distance = 139.0 ± 12.2 cm, *T* = −2.583, df = 6, *p* < 0.05) or Alpine (mean distance = 132.5 ± 10.4 cm, *T* = −2.860, df = 6, *p* < 0.05) traveled, on average, a shorter distance than those in control areas (mean distance = 194.9 ± 24.6 cm) (Figure 3A).

There were also significant differences in velocity between bed bugs in areas treated with CimeXa (mean = 0.46 ± 0.04 cm/s, *T* = −2.582, df = 6, *p* < 0.05) and Alpine (mean = 0.44 ± 0.03 cm/s, *T* = −2.846, df = 6, *p* < 0.05) and those in control areas (mean = 0.65 ± 0.08 cm/s) (Figure 3B).

Insects walking on MotherEarth D treated areas traveled a shorter distance (mean = 146 ± 13.3 cm) and at a lower velocity (mean = 0.49 ± 0.04 cm/s) than those in control areas, but no significant differences were detected (*p* > 0.05) (Figure 3B). MotherEarth D, Alpine and DeltaDust killed 30.0 ± 5.7%, 36.7 ± 8.8% and 30.0 ± 5.7%, respectively, of bed bugs exposed for brief periods of time. Tempo 1% Dust caused intermediate mortality (63.3 ± 12.0%) while higher mortality was observed in bed bugs exposed to Tri-Die Dust (90.0 ± 5.7%). Only bed bugs exposed to CimeXa killed 100% of bed bugs by day 4 post-exposure.

## 4. Discussion

Unlike other studies that evaluate the response of bed bugs to insecticides, the present study has recorded detailed behavioral measurements over a short time frame using a modern video tracking technique. The final choice parameter, a variable that is usually recorded in behavioral studies [25], has limited relevance when capturing information about how bed bugs might react to insecticide deposits that they encounter while they forage for food or as they return to harborages. Our behavioral data support the conclusion that bed bugs avoid some insecticide dusts. The variables that yielded the most informative and consistent results about avoidance behavior toward dusts were elapsed time until first visit to treated areas, number of visits to treated areas, and percent of time spent in treated areas. In turn, distance traveled and velocity were variables used to understand the effect of insecticide dusts on the locomotor activity of bed bugs. Bed bugs took significantly more time to make a first contact with the edge of areas treated with Alpine and MotherEarth D, than control untreated arenas. Likewise, bed bugs visited areas treated with these two dust products less frequently. Similar results were achieved when bed bugs interacted with areas treated with CimeXa. When entering into the treated areas, bed bugs spent less time in areas treated with Tri-Die Dust, CimeXa, Alpine and MotherEarth D, while bed bugs traveled shorter distances, and traveled at slower velocities in areas treated with CimeXa and Alpine. Overall, these results show that bed bugs have behavioral mechanisms to avoid DE, pyrethrins and silica gel-based products. However, responses to insecticides might vary depending on the time the bed bugs have been reared in laboratory conditions [39].

Diatomaceous earth has been perceived as less toxic to mammals and a more sound option for bed bug control [20,40,41]. Several laboratory studies have reported high efficacy of DE against bed bugs [29,31,42]. Diatomaceous earth was more effective against bed bugs when mixed with a dispersal agent (bed bug alarm pheromone components) which enhances locomotor activity of the insects, inducing a higher contact with DE [19]. In forced exposure assays, high doses of DE caused 100% adult mortality after nine [42], and ten days [29] of continuous exposure to DE-treated substrates. In Doggett and Russell [29], DE at a concentration of 1 mg/cm^2^, a similar concentration that induced avoidance in our study, attained 100% adult mortality after 15 days of continuous exposure to treated surfaces. According to Singh et al. [31], confining bed bugs to dust-treated areas does not represent a realistic view of how bed bugs interact with dusts in real infestations. Instead, bed bugs are likely to encounter isolated treated areas and they might be exposed to dusts for only a short time. Studies that simulate short-term exposures in field conditions reported that DE [19,31] or Alpine [31] caused negligible mortality in bed bugs (<20%). In our study, short exposure of bed bugs to DE and Alpine caused low mortality (<36.7%), confirming that brief contact of insects with DE-based products have limited lethal effect. Moreover, one-day exposure to a surface treated with DE or Alpine caused similar low mortality in bed bugs [43]. In this later study, mortality was recorded for only 48 h after exposure, a limited time to observe the insecticidal effects of these products. However, mortality from DE exposure was higher in an experiment where bed bugs interacted with an arena with a DE-treated band on the periphery [31]. Slow or poor performance of DE in laboratory conditions was confirmed in a field study where DE, used as the only-control method, did not reduce bed bug counts in an infested apartment [44]. These results suggest that DE, by itself, is not the best option for bed bug control. Avoidance behavior of bed bugs toward areas treated with DE or DE-based products might compromise the efficacy of these materials even more because it would limit the pickup of dusts. Bed bugs also spent less time in areas treated with Tri-Die Dust, indicating that this formulation has constituents with repellent properties for bed bugs. It is not clear which constituent(s) of the Tri-Die formulation (amorphous silica, piperonyl butoxide, petroleum distillate, or pyrethrins) exerted a repellent effect on bed bugs, although pyrethrins are known to repel some insects [45]. CimeXa and Tri-Die Dust have a high content (91.2% and 40%, respectively) of amorphous silica in their formulations and in both cases, insects spent less time in areas treated with these dusts. It may be possible that dusts that have a high content of silica gel in their formulation may cause a repellent effect on bed bugs but no conclusions may be drawn since the complete formulations of the other dusts are not expressed on the label, in which they may also have high quantities of silica gel.

There is a consensus about the higher efficacy of silica gel-based dust products than other desiccant dusts [30,31,46]. In laboratory studies, CimeXa, at a dose of 1.34 mg/cm^2^, caused substantial mortality (≥95%) within 24 h [46], and similar results were reported by Singh et al. [31]. This fast-killing effect was also observed in bed bugs exposed to smaller quantities of silica gel (0.4 and 0.13 mg/cm^2^) [44]. The efficacy of silica gel applications as a sole treatment was also reported in infested apartments where a substantial reduction in the number of bed bugs (83.2%) was achieved one week post-treatment [46]. Therefore, it is premature to conclude that the avoidance by bed bugs to the silica gel CimeXa, documented here, could potentially hamper the efficacy of treatments. Furthermore, Singh et al. [31] and Potter et al. [46] found that bed bug mortality was high (≥ 95%) after brief contact with CimeXa deposits. We confirmed this with a follow-up experiment where we forcibly exposed bed bugs to CimeXa for an even shorter amount of time than that reported by Singh et al. [31] and Potter et al. [46]. Exposure to CimeXa for 1 min 14 s (the average time bed bugs spent crawling on CimeXa during a 5-min period in our study) was enough to cause 100% mortality. The high lethality of CimeXa was evident when longer exposures to Tri-Die Dust (exposure time = 1 min and 31 s), Tempo 1% Dust (exposure time = 2 min and 8 s) or DeltaDust (exposure time = 2 min and 12 s), caused lower mortality (mean mortality ± SE, 90.0 ± 5.77%, 63.3 ± 12.02%, 30.0 ± 5.77%, for Tri-Die Dust, Tempo 1% Dust, and DeltaDust, respectively). Some bed bugs exposed to formulations containing pyrethroids and/or pyrethrins (Tri-Die Dust, Tempo 1% Dust and Delta Dust) displayed typical neurotoxic effect such as tremors and twitching before death. The above results indicate that avoidance to certain dusts might reduce the efficacy of some insecticides. However, avoidance effects could be overcome by the toxicity of the dust to the bed bugs. Nevertheless, these results provide an early warning about the ability of bed bugs to exhibit behavioral mechanisms that could reduce the impact of insecticide dust treatments. Given the increasing use of these products in structural treatments for pest control [1] development of tolerance to insecticide dust is a concern. Evidence from stored product pests has shown that some strains of *Tribolium castaneum* have become tolerant after continuous exposure to DE [33]. When susceptible strains and tolerant strains were compared, it was reported that tolerant *T. castaneum* avoided DE-treated areas and moved slower, with reduced velocity, through DE-treated wheat grain [33]. Interestingly, in our study, bed bugs displayed similar locomotor responses with CimeXa and the DE-based product Alpine. These responses might be behavioral adaptations that would decrease the pickup of dust particles [33]. In addition, a recent study reported that a multiresistant bed bug population with cuticular thickening exhibited tolerance to sublabel rates of silica gel-based dusts (CimeXa) [41]. These findings are worrisome given the fact that these mechanisms might confer cross resistance across other nonrelated insecticides.

Interestingly, bed bugs did not avoid products that contain pyrethroids (DeltaDust and Tempo 1% Dust). These results are surprising since pyrethroids are known to have repellent properties due to their toxic effects on the peripheral and central nervous systems of insects [47]. Sublethal concentrations of the pyrethroid deltamethrin, the active ingredient in DeltaDust, were avoided and increased locomotor activity in bed bugs [25]. Conversely, Tempo 1% Dust containing the pyrethroid cyfluthrin, did not prevent bed bugs from crossing areas treated with this product [48]. A lack of avoidance behaviors in bed bugs toward pyrethroid-based dust products might be due to low levels of surface exposure to the active ingredient insecticide or a bed bug’s reduced perception of the small dust particles that would normally prevent them from walking on treated substrates.

Behavioral data from our study on Alpine, MotherEarth D, and CimeXa insecticides coincide with results from a previous laboratory study that used an experimental setup to simulate exposure of bed bugs to dusts applied to a perimeter [31]. These authors reported that bed bug nymphs took more time to cross bands treated with Alpine, MotherEarth D, and CimeXa than those who crossed untreated bands. Bed bug responses to MotherEarth D were consistent with those exposed to Alpine and this indicates that DE (the active ingredient present in both formulations at concentrations >95%) is at least one constituent of the formulations responsible for avoidance behaviors observed. Avoidance responses by the blood-feeding bug *Triatoma infestans* to DE depended on the concentration of the dust applied on the substrate [34]. A DE concentration of 1 mg/cm^2^, similar to the concentration used in our study, repelled nymphs of *T. infestans*, whereas lower DE concentrations (0.33 and 0.1 mg/cm^2^) were reported to be attractant and induce settling behavior in nymphs exposed to the treated areas [34]. Although the repellent effects of DE on bed bugs might be due to the chemical properties of the dusts, results reported by Luz et al. [34] suggest that bed bugs avoid high concentrations of dust per area. Diatomaceous earth and Alpine are very lightweight dusts and particles are visible on the substrate even at label rates (Figure 1, video in supplementary files). This might explain why bed bugs did not avoid products with small dust particles such as DeltaDust and Tempo 1% Dust but still caused mortality in short exposure assays. Formulations with small particles are also better able to absorb lipids from an insect’s epicuticle and thus increase insect mortality [49]. In addition, grooming activity could increase exposure to insecticide dusts (see grooming activity in supplementary file in “Tempo 1% Dust”). These findings emphasize that the application of fine layers of dust is essential to reduce avoidance by bed bugs and increase the dust’s effectiveness in field conditions. The presence of the neonicotinoid dinotefuran in Alpine raises the question of whether this insecticide contributes to the avoidance effect observed in bed bugs. There is limited information on the repellent activity of dinotefuran on insects; however, neonicotinoids are generally known as nonrepellent insecticides [50]. Avoidance responses of bed bugs to dusts may be partly responsible for treatment failures that occurred when dusts were used as the only-control method [44]) or when they were combined with other methods [21,51]. High moisture in environments where these desiccant dusts are applied might also reduce the efficacy of dust treatments [52], although waterproof dusts reduce this effect [53].

Insecticides that potentially induce avoidance behaviors in bed bugs could be used in a “push–pull” control strategy if combined with an attractant and a pest control agent. “Push–pull” is a proposed behavior manipulation strategy that has been evaluated successfully in agricultural and livestock pests, as well as *German cockroaches* [54,55]. This strategy consists of displacing insects from a resource (e.g., shelter) and lured with an attractant to areas containing a pest control agent (e.g., traps, nonrepellent insecticides, or baits). Knowledge generated in the last decade on bed bug aggregation pheromones, repellents, attractants, and phagostimulants could be used to develop and validate a push–pull strategy for bed bug control. A possible scenario in a pull–push strategy for bed bugs would be the use of repellent agents such as DEET, isolongifolenone and isolongifolanone, to displace insects from aggregation or hiding sites, and lure them to areas with known bed bug attractants such as CO_2_ and chemical blends [56].

Similarly, products that are avoided by bed bugs could be used in proactive approaches to reduce dispersion of insects within multiunit buildings [57]. A low cost proactive approach based on the application of DE in wall voids was proposed in multiunit facilities [20]. Initial field results are promising; however, further research is needed to validate the benefits of these strategies in different multiunit environments.

## 5. Conclusions

This study demonstrates that bed bugs have behavioral mechanisms that reduce their exposure to some insecticide dusts. These responses might affect the efficacy of bed bug management programs where insecticide dusts are included as a tactic. Nevertheless, the rapid killing effect of some dust insecticides upon brief contact might reduce the impact of avoidance in bed bugs. The study of bed bug responses to insecticide dusts provides not only a better understanding of the effect of dust treatments, but also inspires the development of behavior manipulation strategies that could lead to improved methods for bed bug control.

## Supplementary Materials

The following are available online at www.mdpi.com/2075-4450/8/3/83/s1, Video: https://drive.google.com/drive/folders/0B-kSkIKbE8dhSEpwTzU1ajBPSlE, S1: Interaction between bed bugs and insecticide dusts.
